# Light quality regulates plant biomass and fruit quality through a photoreceptor-dependent HY5-LHC/CYCB module in tomato

**DOI:** 10.1093/hr/uhad219

**Published:** 2023-11-16

**Authors:** Jiarong Yan, Juan Liu, Shengdie Yang, Chenghao Jiang, Yanan Liu, Nan Zhang, Xin Sun, Ying Zhang, Kangyou Zhu, Yinxia Peng, Xin Bu, Xiujie Wang, Golam Jalal Ahammed, Sida Meng, Changhua Tan, Yufeng Liu, Zhouping Sun, Mingfang Qi, Feng Wang, Tianlai Li

**Affiliations:** College of Horticulture, Shenyang Agricultural University, Shenyang 110866, China; College of Horticulture, Shenyang Agricultural University, Shenyang 110866, China; College of Horticulture, Shenyang Agricultural University, Shenyang 110866, China; College of Horticulture, Shenyang Agricultural University, Shenyang 110866, China; College of Horticulture, Shenyang Agricultural University, Shenyang 110866, China; College of Horticulture, Shenyang Agricultural University, Shenyang 110866, China; College of Land and Environment, Shenyang Agricultural University, Shenyang 110866, China; College of Horticulture, Shenyang Agricultural University, Shenyang 110866, China; College of Horticulture, Shenyang Agricultural University, Shenyang 110866, China; College of Horticulture, Shenyang Agricultural University, Shenyang 110866, China; College of Horticulture, Shenyang Agricultural University, Shenyang 110866, China; College of Horticulture, Shenyang Agricultural University, Shenyang 110866, China; College of Horticulture and Plant Protection, Henan University of Science and Technology, Luoyang 471023, China; Henan International Joint Laboratory of Stress Resistance Regulation and Safe Production of Protected Vegetables, Luoyang, 471023, China; College of Horticulture, Shenyang Agricultural University, Shenyang 110866, China; College of Horticulture, Shenyang Agricultural University, Shenyang 110866, China; College of Horticulture, Shenyang Agricultural University, Shenyang 110866, China; College of Horticulture, Shenyang Agricultural University, Shenyang 110866, China; College of Horticulture, Shenyang Agricultural University, Shenyang 110866, China; College of Horticulture, Shenyang Agricultural University, Shenyang 110866, China; Key Laboratory of Protected Horticulture, Ministry of Education, Shenyang 110866, China; College of Horticulture, Shenyang Agricultural University, Shenyang 110866, China; Key Laboratory of Protected Horticulture, Ministry of Education, Shenyang 110866, China

## Abstract

Increasing photosynthesis and light capture offers possibilities for improving crop yield and provides a sustainable way to meet the increasing global demand for food. However, the poor light transmittance of transparent plastic films and shade avoidance at high planting density seriously reduce photosynthesis and alter fruit quality in vegetable crops, and therefore it is important to investigate the mechanisms of light signaling regulation of photosynthesis and metabolism in tomato (*Solanum lycopersicum*). Here, a combination of red, blue, and white (R1W1B0.5) light promoted the accumulation of chlorophyll, carotenoid, and anthocyanin, and enhanced photosynthesis and electron transport rates by increasing the density of active reaction centers and the expression of the genes *LIGHT-HARVESTING COMPLEX B* (*SlLHCB*) and *A* (*SlLHCA*), resulting in increased plant biomass. In addition, R1W1B0.5 light induced carotenoid accumulation and fruit ripening by decreasing the expression of *LYCOPENE β-CYCLASE* (*SlCYCB*). Disruption of *SlCYCB* largely induced fruit lycopene accumulation, and reduced chlorophyll content and photosynthesis in leaves under red, blue, and white light. Molecular studies showed that ELONGATED HYPOCOTYL 5 (SlHY5) directly activated *SlCYCB*, *SlLHCB*, and *SlLHCA* expression to enhance chlorophyll accumulation and photosynthesis. Furthermore, R1W1B0.5 light-induced chlorophyll accumulation, photosynthesis, and *SlHY5* expression were largely decreased in the *slphyb1cry1* mutant. Collectively, R1W1B0.5 light noticeably promoted photosynthesis, biomass, and fruit quality through the photoreceptor (SlPHYB1 and SlCRY1)-SlHY5-*SlLHCA/B/SlCYCB* module in tomato. Thus, the manipulation of light environments in protected agriculture is a crucial tool to regulate the two vital agronomic traits related to crop production efficiency and fruit nutritional quality in tomato.

## Introduction

Plenty of organisms shape their life cycles and activity patterns according to diurnal and seasonal light regime variations. Thus light environments play a critical role in the organization of biological systems ranging from molecules to ecosystems [1, 2]. Light is essential in agricultural production as it is the source of energy for carbon fixation in photosynthesis. Increasing photosynthesis and light capture offers possibilities for improving crop yield and provides a sustainable way to meet the increasing global demand for food.

Plants harvest the useful spectrum for photosynthesis to convert light energy into chemical energy via chlorophylls and carotenoids [3, 4]. Chlorophyll a and b, the critical components of the light-harvesting complex in chloroplasts, absorb the red (R, 600–700 nm) and blue (B, 400–500 nm) portions of sunlight [5]. Carotenoids, an accessory photosynthetic pigment to harvest and transfer light energy to chlorophylls, strongly absorb sunlight in the 400- to 500-nm range [6]. Hence, not all spectral components of sunlight are equally effective for photosynthesis [7].

Plants precisely detect and respond to dynamic changes in light environments via dedicated photoreceptors [8]. The UV RESISTANCE LOCUS 8 (UVR8) receptor detects ultraviolet B light (UV-B; 280–315 nm) [9], while the blue light receptors, including phototropins (PHOTs) [10], cryptochromes (CRYs) [11], and ZEITLUPE family proteins (ZTL, LKP2, and FKF1) [12], monitor blue light (B; 390–500 nm). In addition, the phytochromes (PHYs) are used to perceive far-red (FR; 700–750 nm) and red (R; 600–700 nm) light [13].

Different wavelengths of light influence plant physiological metabolism and development. Previous studies have suggested that B and R light are the most effective light spectra for photosynthesis; however, the greater reflection of green (G) light is the reason for the green appearance of most photosynthetic organisms and leaves [14–16]. Some studies have also shown that G light stimulates photosynthesis by providing carbon gain within shaded canopies [17], and improves drought tolerance by regulating stomatal movement [18]. FR light is reported to attenuate plant photosynthesis [19]. B light regulates the biosynthesis of chlorophyll and stomatal opening, leading to a higher photosynthesis rate [20, 21]. After B light treatment, plant phenolic compounds, such as phenolic acids, phthalic acid, gallic acid, and chlorogenic acid, significantly increased in pea sprouts [22]. B light also largely promotes phenolic substance accumulation in the medicinal plant *Kalanchoe pinnata* [23]. Isorhamnetin, flavonoid, quercetin, and kaempferol in *Ginkgo biloba* were also enhanced by B light [24], and the synergistic enhancement of epidermal flavonols in pepper was observed [25]. However, monochromatic B light causes dwarfing, decreased leaf size and reduced branching [26]. Although R light induces chlorophyll, carotenoid, anthocyanin, and phenolic accumulation, and is the most effective for photosynthesis [15, 27], long-term monochromatic R light usually causes damage to plant growth [20, 28–30]. R light causes leaf curling and inhibits flowering, whereas B light promotes sugar accumulation and fruit development in strawberries [16]. These reports demonstrated that the impacts of monochromatic light on plant growth vary with the plant species and tissue type of plants.

Since long-term monochromatic R or B light has negative effects on normal plant growth, exploring the appropriate R and B combination of light is critical to ensure the healthy growth of plants [20]. For instance, mixed R and B light largely promoted secondary metabolite accumulation in *Artemisia annua* seedlings, including artemisinin and artemisinic acid [31]. Compared with R light, R and B mixed light thickened plant leaves, thus promoting chlorophyll accumulation, photosynthesis, and plant dry weight [28, 32]. In addition, the R:B light ratio of 3:1 improves plant growth and tomato fruit quality [33, 34]. B light induces the accumulation of galantamine and lycoramine in *Lycoris longituba* [35], while mixed R:B light ratios of 1:2 and 2:1 significantly improve anthocyanin and rosmarinic acid contents, respectively, in *Ocimum basilicum* [36]. Moreover, R and B mixed light enhances spinach (*Spinacia oleracea*) and lettuce (*Lactuca sativa*) growth [37]. Thus, different combinations of R and B light have a variety of effects on plant growth, development, and metabolite accumulation among different species.

The roles of FR and G light have been largely neglected in the full light spectrum; nonetheless, these can also stimulate photosynthesis and metabolite accumulation in plants [17, 38, 39]. Therefore, it is essential to explore the appropriate ratios of R and B light mixed with the full spectrum to improve plant growth and metabolite accumulation in tomato. Our data showed that the combination of R, B, and white (W) light [R:W:B = 1:1:0.5 (R1W1B0.5)] promoted the accumulation of chlorophyll, carotenoid, and anthocyanin, and enhanced photosynthesis and electron transport rates by increasing the density of active reaction centers and the transcription of light-harvesting genes, such as *LIGHT-HARVESTING COMPLEX B* and *A* (*SlLHCB* and *SlLHCA*), leading to increased plant biomass accumulation. In addition, R1W1B0.5 light induced carotenoid accumulation and tomato fruit ripening by decreasing the expression of *LYCOPENE β-CYCLASE* (*SlCYCB*). Importantly, disruption of *SlCYCB* largely induced lycopene accumulation in tomato fruit, but significantly reduced chlorophyll content and photosynthesis in tomato leaves, indicating that *SlCYCB* is critical in photosynthesis and fruit metabolism. Molecular studies showed that ELONGATED HYPOCOTYL 5 (SlHY5) directly activates expression of the *SlCYCB*, *SlLHCB*, and *SlLHCA* genes to promote chlorophyll accumulation and enhance photosynthesis rates. In addition, R1W1B0.5 light-induced chlorophyll accumulation, photosynthesis and the transcription of *SlHY5* were largely decreased in the *slphyb1cry1* mutant. Collectively, we identified the molecular mechanisms by which the photoreceptor (SlPHYB1 and SlCRY1)-SlHY5-*SlLHCA/B/SlCYCB* pathway promotes plant photosynthesis, biomass, and fruit quality in tomato in response to R1W1B0.5 radiation.

## Results

### Light environments influence plant pigment and biomass accumulation in tomato

**Figure 1:**
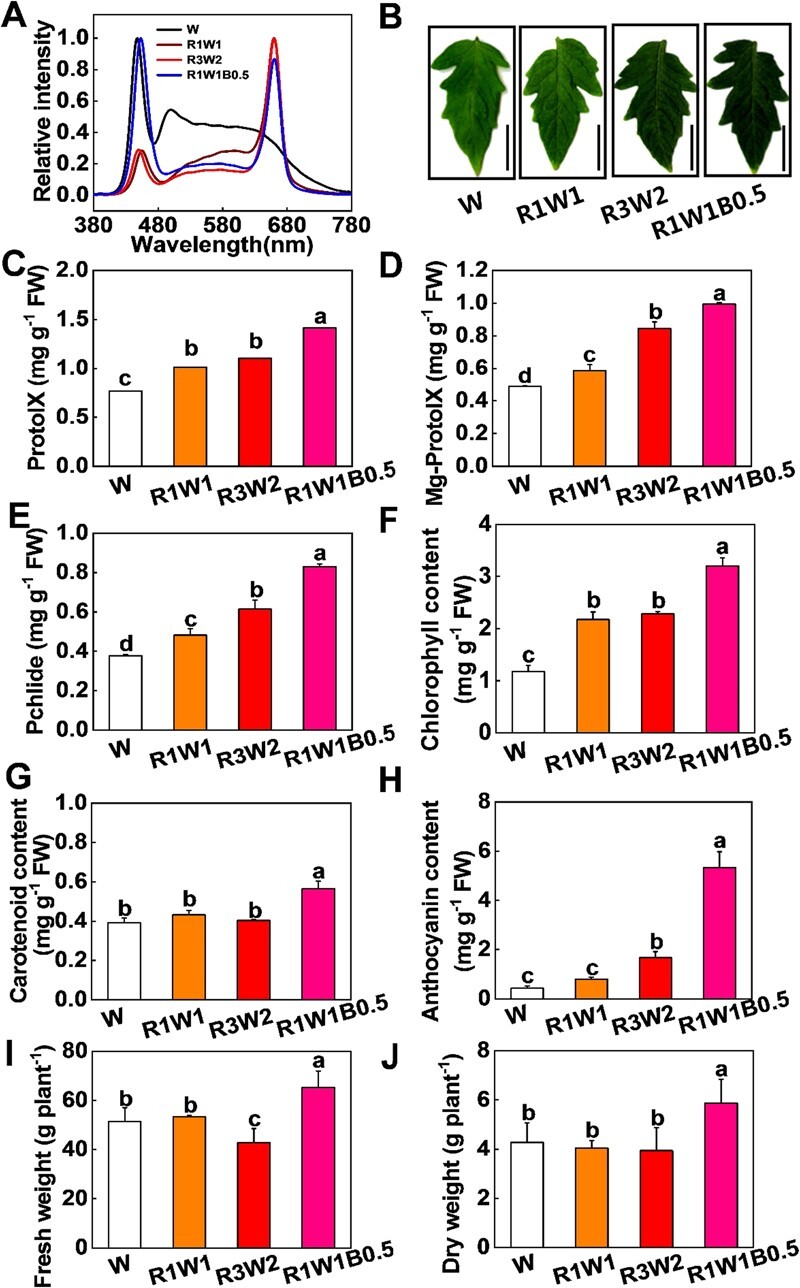
Various light environments influence plant pigment accumulation and biomass in tomato seedlings. **A** Spectrum of different light qualities. **B** Representative images of tomato leaves cultured under white (W), red-white (R1W1 and R3W2), and red-white-blue (R1W1B0.5) light conditions. Scale bar = 2 cm. **C**–**H** ProtoIX (**C**), Mg-ProtoIX (**D**), Pchlide (**E**), chlorophyll (Chl; **F**), carotenoid (**G**), and anthocyanin (**H**) contents in tomato leaves at the five-leaf stage after transfer to various light conditions (W, R1W1, R3W2, and R1W1B0.5) for 15 days. **I**, **J** Fresh weight (**I**) and dry weight (**J**) of plants grown under various light conditions for 15 days. Values are means of three biological replicates (± standard deviation). Statistically significant differences between means are denoted by different letters.

Five-leaf-stage tomato seedlings were transferred to various light conditions (W, R1W1, R3W2, and R1W1B0.5) (Fig. 1A). Tomato plant leaves were dark green when the R light ratio was increased in W conditions [R:W = 1:1 (R1W1) and R:W = 3:2 (R3W2)], especially when B light was added in R1W1 conditions [R:W:B = 1:1:0.5 (R1W1B0.5)] (Fig. 1B). Compared with W conditions, chlorophyll accumulation increased in plant leaves under R1W1 and R3W2 conditions, especially under R1W1B0.5 conditions (Fig. 1F), indicating that increasing the ratio of R light can promote chlorophyll accumulation in tomato. Consistently, the contents of chlorophyll precursors (e.g. Pchlide, Mg-ProtoIX, and ProtoIX) were larger in tomato plants under R1W1, R3W2, and R1W1B0.5 conditions than W conditions (Fig. 1C–E). Strikingly, with increasing R light ratio (from R1W1 to R3W2) under W conditions, Mg-ProtoIX and Pchlide contents gradually increased in tomato leaves, whereas the contents of ProtoIX and chlorophyll showed no changes from R1W1 to R3W2 conditions (Fig. 1C–F). However, the combination of B light and R1W1 (R1W1B0.5) significantly increased ProtoIX and chlorophyll accumulation compared with R1W1 or R3W2 conditions (Fig. 1C–F). Similarly, R1W1B0.5 significantly improved carotenoid and anthocyanin accumulation in tomato leaves compared with plants under W conditions (Fig. 1G and H). Accordingly, the values of tomato seedling biomass were larger under R1W1B0.5 conditions than under W, R1W1, or R3W2 conditions (Fig. 1I and J; Supplementary Data Fig. S1). Our results suggest that an appropriate increase in the proportion of R and B light under W conditions could significantly improve the chlorophyll content and biomass in tomato plants.

### Manipulation of light environments enhances photosynthesis in tomato plants

**Figure 2:**
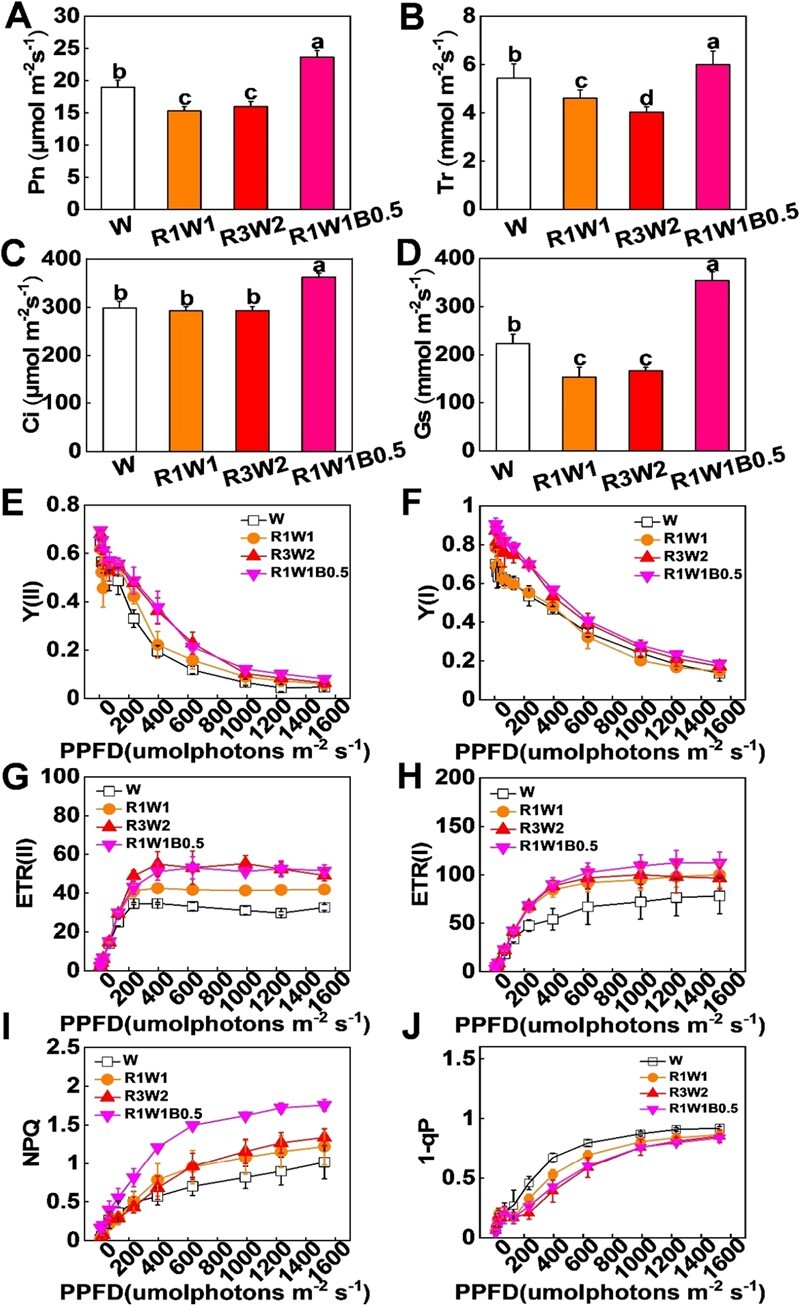
Light quality regulates photosynthesis and electron transport rates in tomato plants. **A**–**D** Net photosynthetic rate (Pn; **A**), transpiration rate (Tr; **B**), intercellular CO_2_ concentration (Ci; **C**), and stomatal conductance (Gs; **D**) in tomato leaves cultured under white (W), red-white (R1W1 and R3W2), and red-white-blue (R1W1B0.5) light conditions for 15 days. **E**–**H** Effective quantum yield of PSII [Y(II); **E**] and PSI [Y(I); **F**], and electron transport rates of PSII [ETR(II); **G**] and PSI [ETR(I); **H**] after tomato exposure to W, R1W1, R3W2, and R1W1B0.5 light treatments for 15 days. **I**, **J** NPQ (**I**) and 1−qP (**J**) after tomato exposure to W, R1W1, R3W2, and R1W1B0.5 light treatments for 15 days. Values are means of three biological replicates (± standard deviation). In **A**–**D**, statistically significant differences between means are denoted by different letters.

Given that chlorophyll contents usually act as an important indicator of photosynthetic rates, we investigated the photosynthesis rate (Pn), intercellular CO_2_ concentration (Ci), stomatal conductance (Gs), and transpiration rate (Tr) of the fifth leaves in tomato plants under different light qualities. We observed that the Pn, Ci, Gs, and Tr for the R1W1B0.5 treatment were obviously higher than for W, R1W1 and R3W2 conditions (Fig. 2A–D). The effective quantum yields of the photosystems [Y(I) and Y(II)] were obviously increased by both R3W2 and R1W1B0.5 light compared with W conditions (Fig. 2E and F). Furthermore, the electron transport rates of the photosystems [ETR(I) and ETR(II)] were increased by R1W1, R3W2 and R1W1B0.5 light treatments compared with W conditions, and the highest values of ETR(II) and ETR(I) were found in plants grown under R1W1B0.5 conditions (Fig. 2G and H). Consistently, plants grown under R1W1, R3W2, and R1W1B0.5 conditions also showed a higher NPQ compared with those grown under W conditions (Fig. 2I). In particular, plants grown under R1W1B0.5 conditions exhibited the highest NPQ. Plastoquinone in its reduced state (1 – qP) had higher values in W light treatments than in R1W1, R3W2, and R1W1B0.5 light treatments (Fig. 2J). Therefore, a larger value of Y(II) was related to the large increase in NPQ and the decrease in 1 − qP in tomato plants grown under R1W1B0.5 conditions.

**Figure 3:**
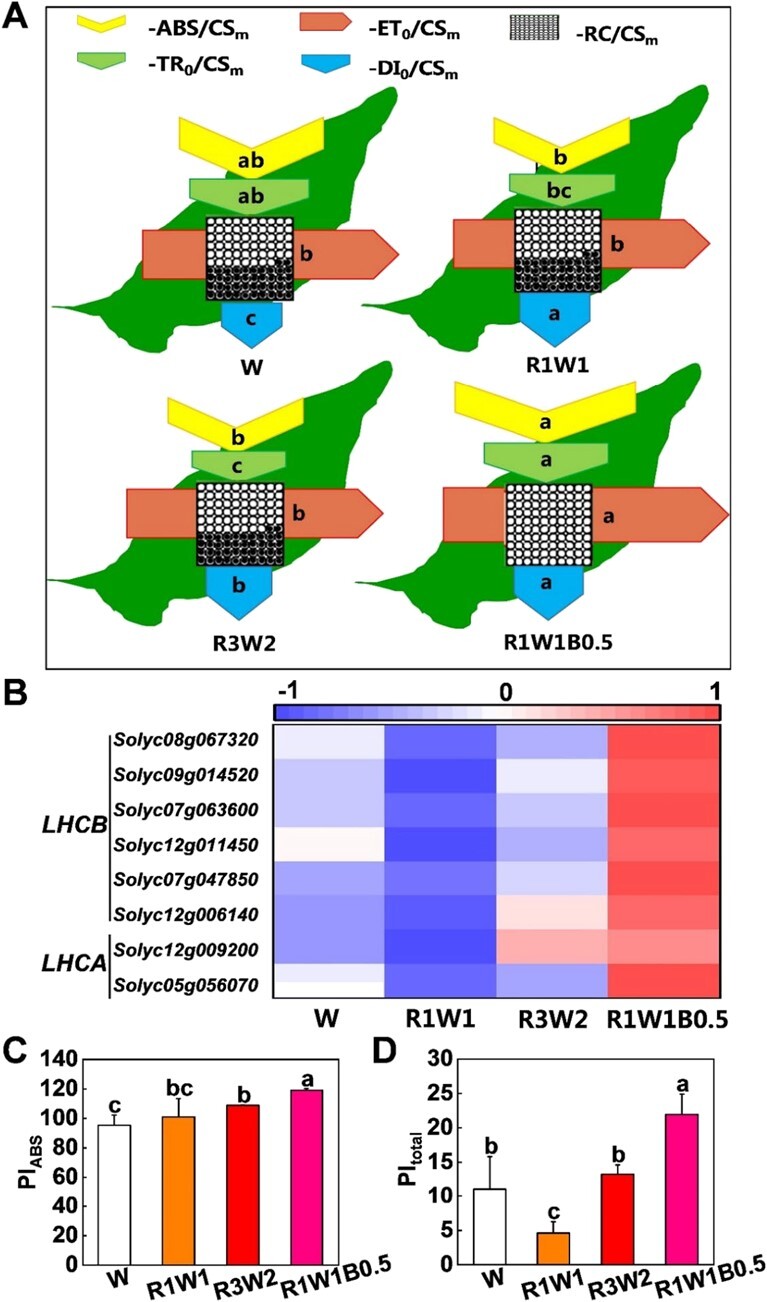
Light quality influences the photosynthetic capacity of tomato plants. **A** Light quality regulates electron absorption, transport, and energy distribution in the photosynthetic response. **B** Expression of *LIGHT-HARVESTING COMPLEX B* and *A* in leaves of tomato plants grown under white (W), red-white (R1W1 and R3W2), and red-white-blue (R1W1B0.5) light conditions for 3 days. **C**, **D** Performance for energy conservation from photons absorbed by PSII to reduction of intersystem electron acceptors (PI_ABS_; **C**) and performance up to the PSI end electron acceptors (PI_total_; **D**) in leaves of tomato plants grown under W, R1W1, R3W2, and R1W1B0.5 light conditions for 15 days. Values are means of three biological replicates (± standard deviation). Statistically significant differences between means are denoted by different letters in **A**, **C**, and **D**.

To further investigate how different light quality treatments regulated PSII activity, leaf energy flux models were constructed (Fig. 3A). Our results showed that R1W1, R3W2 and R1W1B0.5 light treatments significantly increased energy dissipation (DI_0_/CS_m_) (Fig. 3A). In addition, compared with W light treatments, R1W1B0.5 light treatments significantly increased electron transport (ET_0_/CS_m_) (Fig. 3A). Furthermore, the absorption flux (ABS/CS_m_) and trapped energy flux (TR_0_/CS_m_) were higher in tomato leaves under R1W1B0.5 conditions than in those under R1W1 conditions (Fig. 3A). Strikingly, the density of active reaction centers (RCs/CS_m_), as indicated by the number of open circles, and the transcript levels of *LIGHT-HARVESTING COMPLEX B* and A (*SlLHCB* and *SlLHCA*) were also increased by R1W1B0.5 light (Fig. 3A and B). Moreover, plants grown under R1W1B0.5 conditions exhibited the highest performance for energy conservation from photons absorbed by PSII to the reduction of intersystem electron acceptors (PI_ABS_) and performance up to the PSI end electron acceptors (PI_total_) (Fig. 3C and D). These results indicate that R1W1B0.5 light enhances plant photosynthesis by improving the density of active RCs, trapped energy flux of LHCs and photosynthetic electron transport.

### Light environments regulate the metabolism of ripening tomato fruit in a *SlCYCB*-dependent manner

**Figure 4:**
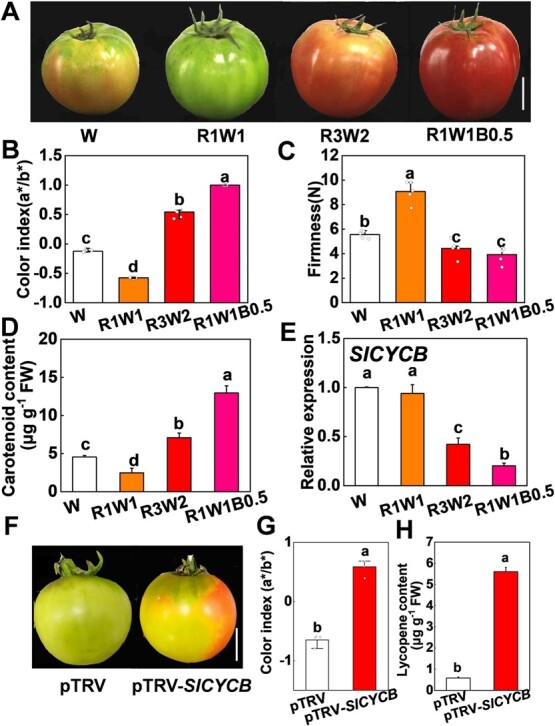
Light quality regulates fruit ripening and metabolism accumulation through an *SlCYCB*-dependent pathway. **A** Representative image of tomato fruits grown under white (W), red-white (R1W1 and R3W2), and red-white-blue (R1W1B0.5) light conditions for 10 days at the mature green stage. Scale bar = 2 cm. **B**–**E** Color index (a*/b*; **B**), fruit firmness (**C**), carotenoid content (**D**), and expression of *SlCYCB* gene (**E**) in tomato fruit after exposure to various light conditions for 10 days. **F** Representative image of tomato fruits in *SlCYCB-*silenced fruits (pTRV-*SlCYCB*) and WT (pTRV) fruits cultured under various light conditions for 5 days. Scale bar = 2 cm. **G**, **H** Color index (a*/b*; **G**) and lycopene contents (**H**) of pTRV-*SlCYCB* and pTRV in tomato fruit cultured for 5 days. Values are means of three biological replicates (± standard deviation). Statistically significant differences between means are denoted by different letters in bar graphs.

Tomato fruit color under R3W2 and R1W1B0.5 light treatments was redder than under W conditions with the higher color index (a*/b* Hunter) and carotenoid, especially under R1W1B0.5 light treatments (Fig. 4A, B, and D). Further, the firmness of tomato fruit under R1W1B0.5 light conditions was lower than under W light treatments (Fig. 4C), which indicated that R1W1B0.5 light accelerated red color development, pigment accumulation, and fruit ripening in tomato. Interestingly, the expression of *LYCOPENE β-CYCLASE* (*SlCYCB*) was obviously lower in fruit under R1W1B0.5 light conditions than under other light conditions (Fig. 4E). To know the role of *SlCYCB* in fruit ripening, we generated *SlCYCB-*silenced fruits (pTRV-*SlCYCB*) (Supplementary Data Fig. S2). *SlCYCB-*silenced fruits appeared more orange-red than wild-type (WT) fruits (pTRV) (Fig. 4F), consistent with the higher color index (a*/b* Hunter) (Fig. 4G). Furthermore, the pTRV-*SlCYCB* fruits accumulated more lycopene than the pTRV fruits (Fig. 4H). Thus, R1W1B0.5 light promotes carotenoid accumulation and fruit ripening by repressing the gene expression of *SlCYCB* in tomato.

### *SlCYCB* promotes chlorophyll accumulation and photosynthesis in response to different light spectra in tomato

**Figure 5:**
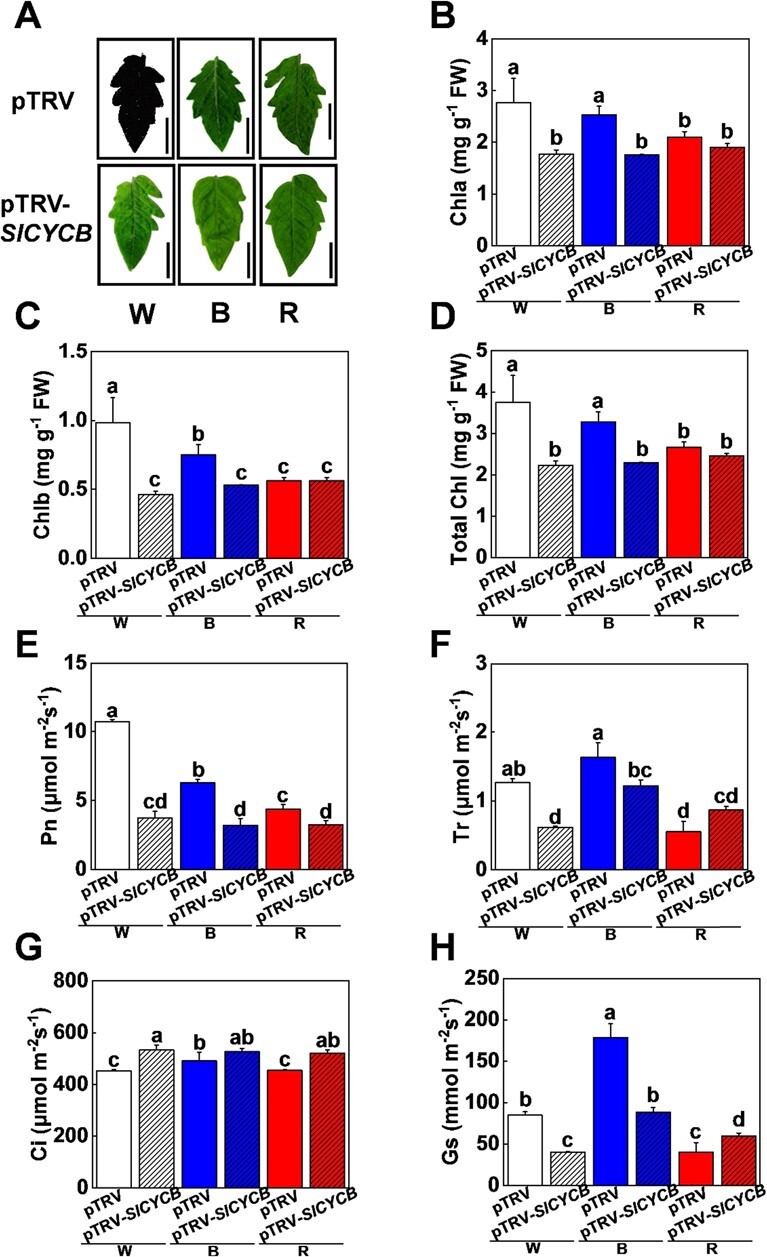
Disruption of *SlCYCB* reduces chlorophyll accumulation and photosynthesis rates in tomato plants under various light conditions. **A** Representative leaf images of *SlCYCB-*silenced plants (pTRV-*SlCYCB*) and WT (pTRV) cultured under white (W), red (R), and blue (B) light conditions for 15 days. Scale bar = 2 cm. **B**–**D** Contents of chlorophyll a (**B**) and b (**C**), and total chlorophyll (**D**) in pTRV-*SlCYCB* and pTRV plants cultured under W, B, and R light for 15 days. **E**–**H** Net photosynthetic rate (Pn; **E**), transpiration rate (Tr; **F**), intercellular CO_2_ concentration (Ci; **G**), and stomatal conductance (Gs; **H**) in pTRV-*SlCYCB* and pTRV tomato plant leaves cultured under W, B, and R light for 15 days. Values are means of three biological replicates (± standard deviation). Statistically significant differences between means are denoted by different letters.

**Figure 6:**
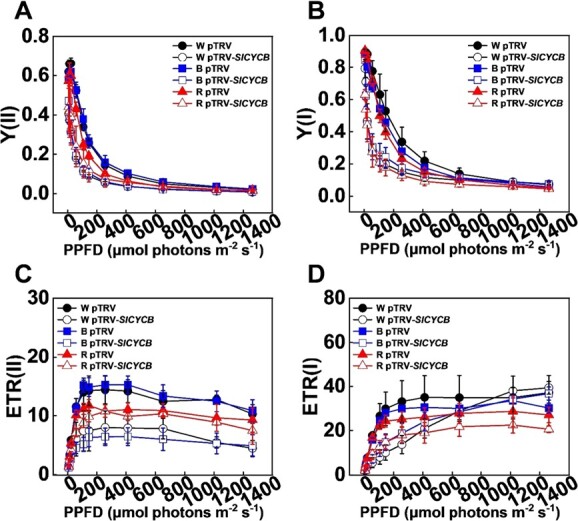
Disruption of *SlCYCB* reduces the effective quantum yield and electron transport rates of PSII and PSI. **A**, **B** Effective quantum yield of PSII [Y(II); **A**] and PSI [Y(I); **B**] in *SlCYCB-*silenced plants (pTRV-*SlCYCB*) and WT (pTRV) after exposure to white (W), red (R), and blue (B) light conditions for 15 days. **C**, **D** Electron transport rates of PSII [ETR(II); **C**] and PSI [ETR(I); **D**] in pTRV-*SlCYCB* and pTRV plants after exposure to white (W), red (R), and blue (B) light conditions for 15 days. Values are means of three biological replicates (± standard deviation).

We next systemically investigated the function of *SlCYCB* in chlorophyll accumulation and photosynthesis in various light quality conditions. Monochromatic R light significantly reduced chlorophyll accumulation, while B light did not change the total chlorophyll content compared with W light (Fig. 5A–D). However, monochromatic R and B light treatments significantly reduced chlorophyll b accumulation and photosynthesis compared with W light (Fig. 5C and E), which suggested that tomato plants are sensitive to monochromatic light, and long-term R or B light could have a large influence chlorophyll accumulation and photosynthesis in tomato plants. Importantly, disruption of *SlCYCB* in tomato plants obviously decreased chlorophyll accumulation in B and W light conditions, but these changes were smaller in R light conditions (Fig. 5A–D). In addition, Pn, Tr, and Gs values decreased in *SlCYCB-*silenced plants (pTRV-*SlCYCB*) compared with WT (pTRV) plants cultivated under W and B light conditions, while Ci increased in pTRV-*SlCYCB* plants cultivated under W, B, and R light conditions (Fig. 5E–H). Furthermore, disruption of *SlCYCB* severely impaired the electron transport rates and effective quantum yield of the photosystem, as indicated by the values of ETR(II), ETR(I), Y(I), and Y(II) (Fig. 6). Together, our results indicate that *SlCYCB* promotes chlorophyll accumulation and photosynthesis in response to various light qualities in tomato.

### SlHY5 acts downstream of SlPHYB and SlCRY1 to promote chlorophyll accumulation and photosynthesis by directly activating *SlLHCs* and *SlCYCB* gene expression

**Figure 7:**
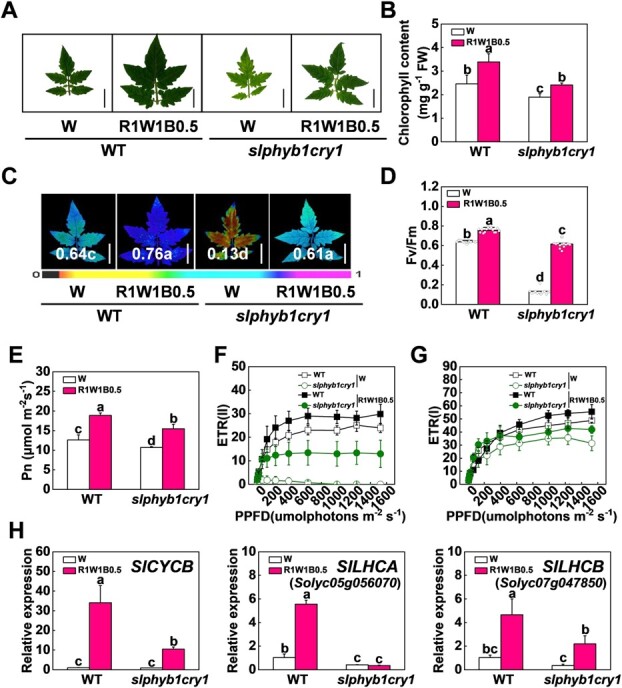
Disruption of *SlPHYB1* and *SlCRY1* reduces chlorophyll accumulation, photosynthesis, and electron transport rates. **A**, **B** Representative tomato leaf images (**A**) and chlorophyll contents (**B**) of *slphyb1cry1* mutant and WT plants cultured under white (W) and red-white-blue (R1W1B0.5) light conditions for 15 days. Scale bar = 2 cm. **C**–**E** Maximum quantum yield of PSII (Fv/Fm; **C**, **D**) and net photosynthetic rate (Pn; **E**) in *slphyb1cry1* mutant and WT plants under W and R1W1B0.5 light conditions for 15 days.Scale bar = 2 cm. **F**, **G** Electron transport rates of PSII [ETR(II); **F**] and PSI [ETR(I); **G**] in *slphyb1cry1* mutant and WT plants under W and R1W1B0.5 light conditions for 15 days. **H** Gene expression of *SlLHCA*, *SlLHCB*, and *SlCYCB* in leaves of *slphyb1cry1* mutant and WT plants cultured under W and R1W1B0.5 light conditions for 3 days. Values are means of three biological replicates (± standard deviation). Statistically significant differences between means are denoted by different letters.

Cryptochromes and phytochromes are the major photoreceptors that perceive blue and red light [11, 13]. To investigate their function in R1W1B0.5 light-induced chlorophyll accumulation and photosynthesis in tomato, we generated *slphyb1cry1* mutants and placed them under W and R1W1B0.5 light conditions. The total chlorophyll content in *slphyb1cry1* mutants significantly decreased compared with WT plants (Fig. 7A and B). Meanwhile, the photosynthesis and electron transport rates of the photosystem were lower in the *slphyb1cry1* mutant than in WT, as indicated by the values of Pn, Fv/Fm, ETR(I), ETR(II), Y(II), and Y(I) (Fig. 7C–G; Supplementary Data Fig. S3). These results indicate that SlPHYB1 and SlCRY1 positively regulate chlorophyll accumulation and photosynthesis in tomato plants. In addition, we found that R1W1B0.5 light increased the values of chlorophyll content, Pn, Fv/Fm, ETR(I), ETR(II), Y(II), and Y(I) in WT, but these effects were were almost abolished in *slphyb1cry1* mutants (Fig. 7A–G; Supplementary Data Fig. S3). Meanwhile, R1W1B0.5 light induced the transcription of *SlLHCA* (Solyc05g056070), *SlLHCB* (Solyc07g047850), and *SlCYCB* in WT plants, but these effects were mostly abolished in the *slphyb1cry1* mutant plants (Fig. 7H). These results indicate that R1W1B0.5 light-induced chlorophyll accumulation and photosynthesis is dependent on SlPHYB1 and SlCRY1 in tomato plants.

**Figure 8:**
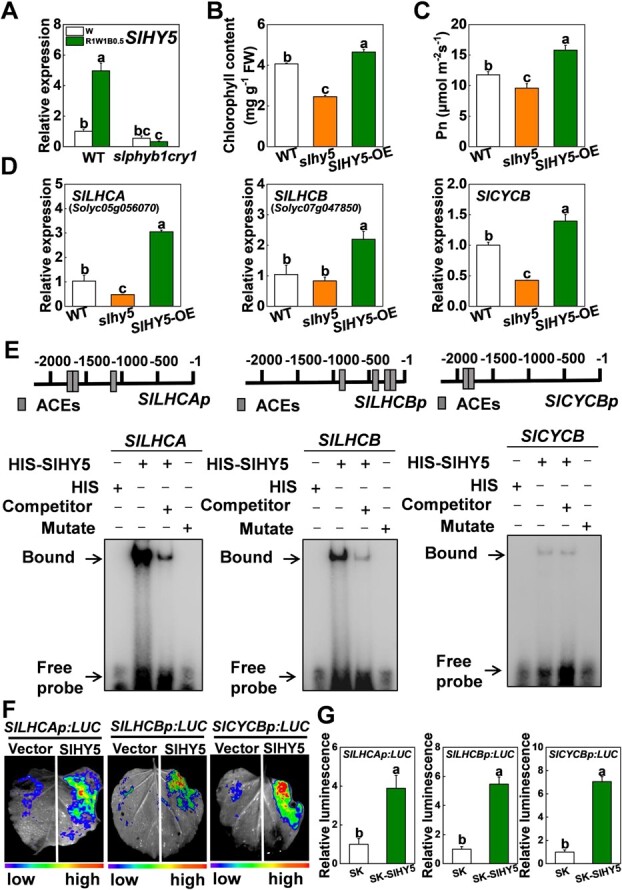
SlHY5 directly activates *SlLHCA*, *SlLHCB*, and *SlCYCB* to promote chlorophyll accumulation and photosynthesis. **A***SlHY5* gene expression in *slphyb1cry1* mutant and WT plants grown under white (W) and red-white-blue (R1W1B0.5) light conditions for 3 days. **B**, **C** Chlorophyll contents (**B**) and net photosynthetic rate (Pn; **C**) in WT, *SlHY5*-overexpressing plants (*SlHY5*-OE), and *slhy5* mutants cultured under R1W1B0.5 light conditions for 15 days. **D** The transcript levels of *SlLHCA*, *SlLHCB*, and *SlCYCB* in leaves of WT plants and *SlHY5*-OE and *slhy5* mutants cultured under R1W1B0.5 light conditions for 3 days. **E** EMSA of SlHY5 associated with *SlLHCA*, *SlLHCB*, and *SlCYCB*. **F**, **G** Dual-luciferase assay for SlHY5 regulation of the expression of *SlLHCA*, *SlLHCB*, and *SlCYCB*. Values are means of three biological replicates (± standard deviation). Statistically significant differences between means are denoted by different letters.

Interestingly, the transcript level of *SlHY5* was significantly increased in WT plants grown under R1W1B0.5 light conditions, while the expression of *SlHY5* was not further enhanced in *slphyb1cry1* mutant plants grown under R1W1B0.5 light conditions (Fig. 8A). This suggests that *SlHY5* works downstream of *SlPHYB1* and *SlCRY1*, and may be critical for R1W1B0.5 light induction of chlorophyll accumulation and photosynthesis in tomato. To confirm this, we observed the chlorophyll content and Pn in WT, *SlHY5*-overexpressing (*SlHY5*-OE) plants, and *slhy5* mutants. Compared with WT, chlorophyll content and Pn were significantly decreased in *slhy5* mutants, whereas they were increased in *SlHY5*-OE plants, which indicated that SlHY5 promotes chlorophyll content and Pn in tomato plants (Fig. 8B and C). Furthermore, the expression levels of *SlLHCA*, *SlLHCB*, and *SlCYCB* significantly decreased in *slhy5* mutants, but showed a large increase following *SlHY5* overexpression (Fig. 8D), which indicated that SlHY5 positively regulates *SlLHCA*, *SlLHCB*, and *SlCYCB* gene expression. PlantCARE analysis suggested that the *SlLHCA*, *SlLHCB*, and *SlCYCB* promoters have potential ACGT-containing elements (ACEs) for SlHY5 (Fig. 8E). EMSA showed that SlHY5 protein significantly reduced the migration of probes containing the ACEs from the *SlLHCA*, *SlLHCB*, and *SlCYCB* promoters, but had no effect on the mutant probes. In addition, the binding of SlHY5 to the *SlLHCA*, *SlLHCB*, and *SlCYCB* promoters was further verified by dual-luciferase assays. Compared with the control, SlHY5 enhanced the activities of the *SlLHCA*, *SlLHCB*, and *SlCYCB* promoters (Fig. 8F and G). Together, these results indicate that SlHY5 acts downstream of SlPHYB1 and SlCRY1 to regulate the expression of *SlLHCA*, *SlLHCB*, and *SlCYCB* by directly binding the *cis*-acting element of these target genes’ promoters, promoting chlorophyll accumulation and photosynthesis in tomato plants under R1W1B0.5 light conditions.

## Discussion

Light is the source of energy for carbon fixation in photosynthesis. Increasing photosynthesis and light capture offers possibilities for improving crop yield and provides a sustainable way to meet the increasing global demand for food. However, vegetation shade and the low transmittance of plastic film seriously affect the light environment in greenhouses and reduce photosynthesis and yield in vegetable crops. Here we demonstrated that managing light quality (photo spectrum) for photosynthesis offers the possibility of increasing crop production in protected horticulture. The combination of W, R, and B light (R1W1B0.5) allowed us to harvest 26.93 and 37.15% more fresh weight (FW) and dry weight (DW) than in W light conditions (Fig. 1I and J). DW and FW were highest in tomatoes grown under R1W1B0.5 treatments, but there were no changes in DW of plants grown under W, R1W1, and R3W2 (Fig. 1I and J; Supplementary Data Fig. S1). Moreover, compared with R1W1, R3W2 reduced FW in tomato plants (Fig. 1I; Supplementary Data Fig. S1A and C), which indicated that excessive increases in R light could even reduce plant biomass. Consistently, it has been observed that monochromatic R light inhibits cucumber FW and DW compared with various R and B light combinations or R, G, and B light combinations [40]. In addition, increased R light proportion enhanced petiole distortion in lettuce [41], but B increased the DW of oyster mushrooms [42]. Hence, the appropriate ratios of R and B light are critical for plants’ healthy growth. Studies have also shown that mixed R and B light improves the growth of spinach, tomato, and lettuce, but the appropriate R/B ratio varies among species [28, 37].

Not all spectral components of sunlight are equally effective for photosynthesis [7]. Although the primary molecular pigments absorb largely the R and B portions of sunlight [43], long-term monochromatic light treatment would reduce photosynthetic capacity. Compared with W light, monochromatic R light greatly decreases photosynthesis in cucumber and pepper [20, 44, 45]. Consistently, our results showed that the value of Pn was decreased in plants under R1W1 and R3W2, while it was increased in plants under R1W1B0.5 conditions compared with W conditions (Fig. 2A), indicating that the optimal light spectrum combination is crucial for improving photosynthesis. Consistently, we also found that R1W1B0.5 light treatments largely induced chlorophyll and carotenoid accumulation compared with W light treatments (Fig. 2C–G). Previous studies have shown that B light can work together with R light in chlorophyll and carotenoid accumulation. For example, B light not only induces the transcription of chlorophyll biosynthesis genes (such as *MgCH*, *FeCH*, and *GluTR*) [46], but also promotes the accumulation of 5-aminolevulinic acid (ALA) [47]. R light also promotes chlorophyll accumulation in pepper, lettuce, kale, and basil [37]. In addition, high B/R ratios greatly increase carotenoid accumulation [37].

The PSII, which consists of a *β*-carotene- and chlorophyll a-binding dimeric core complex, forms supercomplexes for photochemical reactions with the antenna system [48, 49]. Our results showed that R1W1B0.5 light improves the effective quantum yield of the photosystem by increasing the density of active RCs, energy dissipation and electron transport rates, as evidenced by RCs/CS_m_, ETR(II), ETR(I), ET_0_/CS_m_, NPQ, and DI_0_/CS_m_ (Figs 2E–J and 3A). The highest ET_0_/CS_m_ was found in plants under R1W1B0.5 treatments, due to higher repression of re-oxidation of Q_A_^−^ to Q_A_. Antennas are arranged into an inner layer of monomeric light-harvesting complex (LHC) proteins and an outer layer of trimeric LHCII subunits [50]. Here, our results showed that the transcription of *SlLHCB* and *SlLHCA* was obviously induced by R1W1B0.5 light treatments (Fig. 3B). PI_ABS_ and PI_total_ represent the function of PSII and amalgamate the energy fluxes from the early absorption process until plastoquinone reduction and performance up to the PSI end electron acceptors, respectively [51]. Here, the values of PI_ABS_ and PI_total_ were high in plants grown in R1W1B0.5 light conditions, indicating that an optimal increase in R and B light in the presence of W light could improve the performance of PSI and PSII in tomato plants (Fig. 3C and D). In conclusion, our results suggest that R1W1B0.5 light improves the performance of photosynthesis by promoting photosynthetic pigment accumulation, increasing the density of active RCs, elevating electron transport, and enhancing energy dissipation in tomato plants.

It is of crucial importance to explore the optimal light combination that can boost both biomass and quality. We show that R1W1B0.5 light treatments also promoted tomato fruit ripening and carotenoid accumulation (Fig. 4A–D). We observed that R1W1B0.5 light can repress *SlCYCB* gene expression in tomato fruit (Fig. 4E). Moreover, disruption of the *SlCYCB* gene significantly accelerated tomato fruit ripening and lycopene accumulation (Fig. 4F–H). These observations suggest that R1W1B0.5 light greatly improved fruit ripening and lycopene accumulation by reducing the gene expression of *SlCYCB* in tomato fruit. In *slcrtl*-overexpressing tomato plants, lycopene *β*-cyclase (SlCYCB) enzyme activity and *SlCYCB* expression were increased, which resulted in conversion of *trans*-lycopene into *β*-carotene [52–54]. In addition, *slcrtl* also regulates *β*-ring-derived xanthophylls by promoting lycopene *β*-cyclase accumulation [55]. This change in *β*-ring-derived xanthophylls could also affect chlorophyll accumulation because of their association with photosynthetic complexes [54]. Our results showed that disruption of the *SlCYCB* gene reduced chlorophyll accumulation and photosynthesis in tomato plants in W, R, and B light conditions (Fig. 5). Furthermore, the electron transport rates and effective quantum yield were significantly lower in *SlCYCB*-silenced plants compared with those in WT under W, R, and B light (Fig. 6). Therefore, the carotenoid pathway may also influence photosynthetic efficiency, leading to activation of several retrograde signals or sugar signals to affect plant growth and metabolism.

Light is a major regulator for chloroplast biogenesis and chlorophyll biosynthesis. Plants utilize various photoreceptors, such as cryptochromes and phytochromes, to reduce shade-induced leaf senescence. Here, we found that R and B light-enhanced chlorophyll accumulation and photosynthetic efficiency in WT were significantly decreased in *slphyb1cry1* mutants (Fig. 7A–G). Consistently, phyB promotion of chlorophyll biosynthesis and other photosynthetic pigments has been shown in *Arabidopsis* [56]. B light delays leaf senescence in WT but not in *Atcry1cry2* mutants [57]. Furthermore, the lower content of chlorophyll and photosynthetic efficiency in *slphyb1cry1* mutants were accompanied by strong downregulation of genes encoding subunits of light-harvesting complexes: SlLHCA and SlLHCB (Fig. 7H). A recent study has emphasized a genome-wide role of phytochromes and cryptochromes in the regulation of the chloroplast, including genes in both the plastid and the nucleus, whose products act in plastid development, the production of plastid essential metabolites, and the onset of photosynthesis [58]. Taking these findings together, it can be concluded that the induction of chlorophyll accumulation and photosynthetic efficiency is mediated by phytochromes and cryptochromes in response to R and B light.

**Figure 9:**
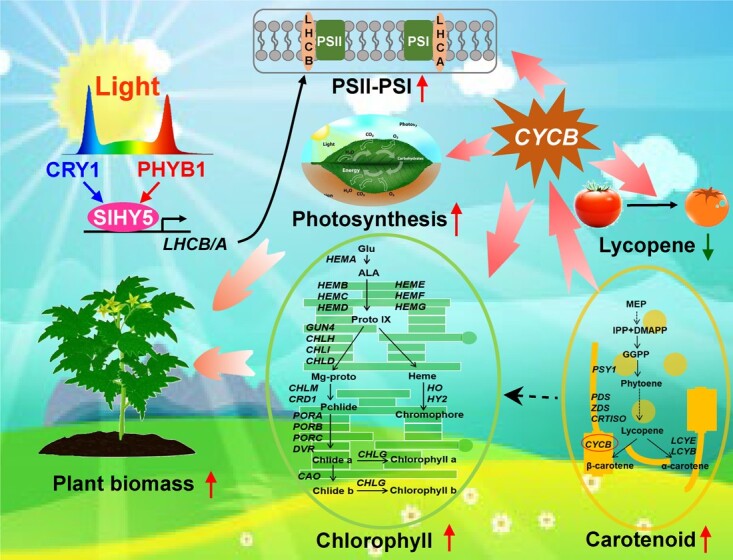
A proposed model of light quality regulation of photosynthesis and fruit metabolism in tomato. Manipulation of light environments promotes the accumulation of chlorophyll, carotenoid, and anthocyanin, and enhances photosynthesis and electron transport rates by increasing the density of active reaction centers and the expression of *LIGHT-HARVESTING COMPLEX B* and *A*, resulting in increased plant biomass in tomato. In addition, R1W1B0.5 light induces fruit ripening and carotenoid accumulation by decreasing the expression of *LYCOPENE β-CYCLASE* (*SlCYCB*). In brief, R1W1B0.5 light noticeably promotes photosynthesis, biomass, and fruit quality through the photoreceptor (SlPHYB1 and SlCRY1)-SlHY5-*SlLHCA/B/SlCYCB* module in tomato.

After R and B light absorption, phytochromes and cryptochromes repress CONSTITUTIVE PHOTOMORPHOGENIC 1 (COP1)-dependent degradation of HY5 [59]. Furthermore, R and B light enhances HY5 protein accumulation by inducing the transcription of *SlHY5* (Fig. 8A) and its protein phosphorylation [60]. HY5 regulates photopigment accumulation and photosynthetic efficiency in response to light through five main mechanisms. First, HY5 regulates the expression of chlorophyll biosynthesis genes, such as *GENOMES UNCOUPLED 5* (*GUN5*) and *PCHLIDE OXIDOREDUCTASE C* (*PORC*), and components of the light-harvesting complex, such as *AtLHCA4*, *AtLHCB1.1*, and *AtLHCB1.3* [61, 62]. Similarly, we found that SlHY5 directly activated *SlLHCA* and *SlLHCB* expression via binding to their promoters (Fig. 8D–G). Second, HY5 directly regulates the expression of *GOLDEN LIKE2* (*GLK2*) [63], which acts as a positive regulator in the expression of nuclear photosynthetic and chlorophyll biosynthetic genes, in particular *CHLIDE A OXYGENASE* (*CAO*), *MAGNESIUM CHELATASE ENCODING GENE 26* (*CHL26*), *MAGNESIUM CHELATASE* (*CHLH*), and *GLU-tRNA REDUCTASE 1* (*HEMA1*) [64]. Third, HY5 promotes the expression of *DIGALACTOSYLDIACYLGLYCEROL SYNTHASE 1* (*DGD1*) for chloroplast biogenesis [65]. Fourth, HY5 regulates chloroplast transcription by a nuclear-encoded sigma factor, *SIGMA FACTOR5* (*SIG5*) [66, 67]. Fifth, HY5 directly associates with and activates *STOMAGEN*, which in turn stabilizes SPEECHLESS (SPCH) in the epidermis, resulting in the promotion of stomatal production [68]. Since mesophyll cells are the workhorses for photosynthesis, this HY5–STOMAGEN module likely enables these cells in the inner tissue to signal stomatal production on the epidermis for carbon uptake when they are activated by light.

### Conclusions

Our results suggest that a mixture of red, blue, and white light (R1W1B0.5) is more effective than monochromatic R, B, or W light in terms of promoting pigment accumulation, photosynthesis, plant biomass, and fruit ripening in tomato (Fig. 9). Notably, R1W1B0.5 light induces chlorophyll accumulation and photosynthesis through phytochrome- and cryptochrome- dependent pathways in tomato plants. Compared with WT, the chlorophyll content and photosynthesis were largely decreased in the *slphyb1cry1* mutant. Furthermore, the transcription of *SlHY5* was significantly decreased in the *slphyb1cry1* mutant. EMSA and dual-luciferase assay indicated that SlHY5 directly associates with the promoters of *SlLHCA*, *SlLHCB*, and *SlCYCB* and activates their expression, subsequently promoting chlorophyll accumulation and photosynthesis. Thus, manipulation of these genes and artificial light environments can be promising strategies to improve biomass and fruit quality in tomato.

## Materials and methods

### Plant materials and growing conditions

We got *slphyb1cry1* mutants in the cv ‘Moneymaker’ background from the Tomato Genetics Resource Center (http://tgrc.ucdavis.edu). The *slhy5* mutant and *SlHY5*-OE plants in the cv ‘Ailsa Craig’ background were obtained as previously [69–71]. Seedlings were cultivated under a 12-h light/25°C and 12-h dark 20°C cycle at 200 μmol m^−2^ s^−1^ with 65% humidity. The *SlCYCB-*silenced plants were obtained as previously reported [72–74]. The *SlCYCB* complementary DNA fragment was PCR-amplified with the gene primers shown in Supplementary Data Table S1. We digested the *SlCYCB* amplified fragment with XbaI/BamHI and cloned it into pTRV2 vector. The resulting construct was transformed into *Agrobacterium tumefaciens* strain GV3101. We mixed the *A. tumefaciens* of the pTRV1 and pTRV2 target genes (or pTRV2 for the controls) in a 1:1 (v/v) ratio. The infiltration solution was injected into the leaves of 15-day-old tomato seedlings as reported earlier [72, 75]. The infiltration solution (1-ml syringe) was introduced into the mature green stage of tomato fruits via the stylar apex [76, 77]. RNAs of infiltrated fruits and leaves were collected 5 and 30 days later, respectively, and we then tested gene silencing efficacy by RT–qPCR methods. For VIGS experiments, plants were placed under a 12-h light/dark cycle with a 200 μmol m^−2^ s^−1^ PPFD and a 21°C temperature condition.

### Light treatments

Illumination conditions were as follows: red:white light = 1:1 (R1W1), red:white light = 3:2 (R3W2), red:white:blue light = 1:1:0.5 (R1W1B0.5), and white (W) light as a control (Fig. 1A). Light intensity (PPFD) was 200 μmol m^−2^ s^−1^. R light (λ_max_ = 660 nm) and B light (λ_max_ = 460 nm) were used to adjust the R:W:B ratios. We used a Lighting Passport (Asensetek Inc., China) to test the light intensity and light spectrum. At the five-leaf stage, WT and gene-silenced plants were placed in different light conditions (W, R1W1, R3W2, and R1W1B0.5) for 15 days. Moreover, in the case of the fruit experiment, plants at the mature green stage of tomato fruits were transferred to different light conditions (W, R1W1, R3W2, and R1W1B0.5) and the light treatments lasted for 10 days. Fruit gene-silencing efficacy was evaluated 5 days after the infiltration of the mature green-stage fruits. After being confirmed by RT–qPCR, the gene-silenced tomato fruits were transferred to R1W1B0.5 light conditions for 5 days.

### Measurements of pigment contents

Carotenoids and chlorophyll contents in leaves were determined according to the previously described protocol [78]. Briefly, 0.3 g fresh healthy leaves of tomato seedlings were weighed and put into a mixture of 5 ml 80% acetone and 5 ml ethanol, then shaken for 24 h in darkness. The carotenoid and chlorophyll contents were determined with absorption at 663, 645, and 470 nm in clear supernatants using a UV–Visible Spectrophotometer (Cary 50, Varian, CA, USA).

ProtoIX, Mg-ProtoIX, and Pchlide were measured with minor modifications [79]. Fresh leaves (0.5 g) of tomato were homogenized in 25 ml of ice-cold acetone:0.1 mol l^−1^ NH_4_OH (8:2 v/v)/ml reaction mixture, and incubated overnight at 4°C under dark conditions. After 4°C centrifugation (12 000 *g*) for 10 min, absorptions at 628 nm (OD628), 590 nm (OD590) and 575 nm (OD575) were examined in a spectrophotometer. ProtoIX, Mg-ProtoIX, and Pchlide contents were calculated using the following formulae: C_ProtoIX_ = 0.18016 × OD575–0.04036 × OD628–0.04515 × OD590; C_Mg-ProtoIX_ = 0.06077 × OD590–0.01937 × OD575–0.003423 × OD628; and C_Pchlide_ = 0.03563 × OD628 + 0.007225 × OD590–0.02955 × OD575.

The anthocyanins in tomato leaves were extracted according to the previously described protocol with minor modifications [80]. Frozen leaf (0.1 g) was ground to powder and placed in 1 ml methanol:acetic acid (99:1, v:v) at 4°C overnight. After centrifugation at 13 400 *g*, absorption at 530, 620, and 650 nm of the clear supernatants was examined with a spectrophotometer. The anthocyanin content was measured as absorbance = [(A_530_ − A_650_) − 0.2 × (A_650_ − A_620_)]/0.2.

Lycopene was extracted from fruits and determined with a previously described method with minor modifications [81]. Tomato freeze-dried pericarp tissue sample (0.5 g) was mixed with 5 ml hexane:acetone:ethanol (2:1:1, v/v/v) and homogenized for 1 min. After homogenization, we added 1.5 ml water and performed a 10-s vortex of the sample. The lycopene content was examined based on absorption at 503 nm of the organic phase (hexane) after phase separation on ice with the UV–Visible Spectrophotometer (Cary 50, Varian, CA, USA). Results were represented as μg g^−1^ FW.

### Gas exchange parameters and fresh and dry weights

The net CO_2_ assimilation rate (Pn) was examined on the fifth leaf using an LI-6400 (LI-COR, Inc., Lincoln, NE, USA) [72, 74]. The CO_2_ concentration and air flow rate of the leaf chamber were 400 μmol s^−1^ and 500 μmol mol^−1^, respectively. The relative air humidity, leaf temperature and PPFD were 85%, 25°C, and 630 μmol m^−2^ s^−1^, respectively.

After 15 days of various light treatments, tomato plants were harvested and measured as FW (g). Plants were oven-dried at 80°C for 5 days to measure the DW (g).

### Chlorophyll fluorescence determination

OJIP curves were examined with a Dual-PAM-100 (Heinz Walz, Effeltrich, Germany), and the JIP test parameters were analyzed as previously described [69, 75, 82]. After 30 min of dark adaptation the electron transport rates [ETR(II) or ETR(I)], effective quantum yield of photosystems [Y(II) and Y(I)], photochemical quenching coefficient (qP), maximum quantum yield of PSII (Fv/Fm), and energy dissipation of PSII (NPQ) were determined in plants [69, 73, 75].

### Assessment of fruit color and firmness

Fruit color was investigated with a Konica Minolta CR-400 colorimeter (Konica Minolta Sensing, Inc., Osaka, Japan) in the CIE mode L*, a*, and b* as previously described [83]. The L* values represent lightness, a* values represent the green–red, and b* values represent the blue–yellow color components. Coloration was calculated as the a*/b* Hunter ratio. We randomly selected six fruits from each treatment and assessed four locations around the equatorial plane of the fruit.

Fruit firmness was determined using a fruit texture analyzer (CT3, Brookfield Inc., Middleboro, USA) equipped with a 2-mm diameter probe by inserting it into the fruit at a depth of ~7 mm. Firmness was recorded twice at the equator of each fruit, the two measurements being taken at 90° to each other.

### RT–qPCR analysis

Total RNA was extracted by using an RNAprep Pure Plant Kit (Tiangen Biotech, Beijing, China). Complementary DNA (cDNA) was synthesized with the ReverTra Ace qPCR RT Kit (Toyobo, Osaka, Japan). The SYBR Green PCR Master Mix Kit (TaKara Bio Inc., Kusatsu, Japan) and an Applied Biosystems 7500 device (qTower3G, Jena, Germany) were used for RT–qPCR analysis [70, 71, 84]. *ACTIN2* was used as a reference. Primers are listed in Supplementary Data Table S2.

### Electrophoretic mobility shift assay

The EMSA was carried out with recombinant SlHY5-His protein purified from *Escherichia coli* BL21*.* The LightShift Chemiluminescent EMSA kit (cat. no. 20148; Thermo Fisher, USA) was used for EMSA as previously described [69–71, 84]. Primers are listed in Supplementary Data Table S3.

### Dual-luciferase assay

The CDS of SlHY5 was cloned and inserted into the pGreenII-0029-62-SK vector to form an effector, and the promoters of *SlLHCA*, *SlLHCB*, and *SlCYCB* were ligated into pGreenII-0800-LUC vector to form reporters. The primers used are listed in Supplementary Data Table S1. The paired effector and reporter were co-transfected into *Nicotiana benthamiana* leaves*.* Images were collected with a Night Shade LB 985 system (Berthold) after 3 days, as previously described [71].

### Statistical analysis

Experiments were conducted in a completely randomized design. Data analyses were performed using ANOVA followed by Tukey’s test with SPSS software (IBM Corp., Armonk, NY, USA). Differences were considered significant at a *P*-value <.05.

## Acknowledgements

We thank the Tomato Genetics Resource Center (http://tgrc.ucdavis.edu) for offering *slphyb1cry1* mutant, ‘Moneymaker’, and ‘Ailsa Craig’ seeds. This work was funded by the National Natural Science Foundation of China (32122081, 32272698), the National Key Research and Development Program of China (2023YFF1002000), the Natural Science Foundation of Liaoning Province for Excellent Youth (2022-YQ-18), the National Key Research and Development Program of China (2019YFD1000300), the China Agriculture Research System (CARS-23), the National Natural Science Foundation of China (31801904, 31991184), the Liao Ning Revitalization Talents Program (XLYC1807020), the Young and Middle-aged Science and Technology Innovation Talent Support Program in Shenyang (RC200449), the Ministry of Science and Technology of the People’s Republic of China (DL2022026004L), and the Innovative Research Team (Science and Technology) in University of Henan Province (23IRTSTHN024).

## Author contributions

T.L. and F.W. conceived and planned the research. J.Y., J.L., S.Y., C.J., Y.L., N.Z., Y.Z., X.S., K.Z., Y.P., X.W., and X.B. conducted the experiments. J.Y., Z.S., X.S., M.Q., S.M., C.T., T.L., Y.L., and F.W. analyzed the data. J.Y. and F.W. wrote the article. F.W., T.L., and G.A. revised the article. All authors read and approved the ultimate manuscript.

## Data availability

All relevant data in this study are provided in the article and its supplementary files. All data and material reported in this manuscript are available from the corresponding author upon request.

## Conflict of interest

The authors declare that they have no competing interest.

## Supplementary data

Supplementary data is available at *Horticulture Research* online.

## Supplementary Material

Web_Material_uhad219
