# Supplementary material for: Light quality regulates plant biomass and fruit quality through a photoreceptor-dependent HY5-LHC/CYCB module in tomato
Source: Hortic Res. 2023 Nov 16;10(12):uhad219. doi: 10.1093/hr/uhad219 (PMC10699845; doi:10.1093/hr/uhad219)
Supplement: Web_Material_uhad219 [file web_material_uhad219.zip › 2023.10.4.Supplemental figures 1-3.docx]

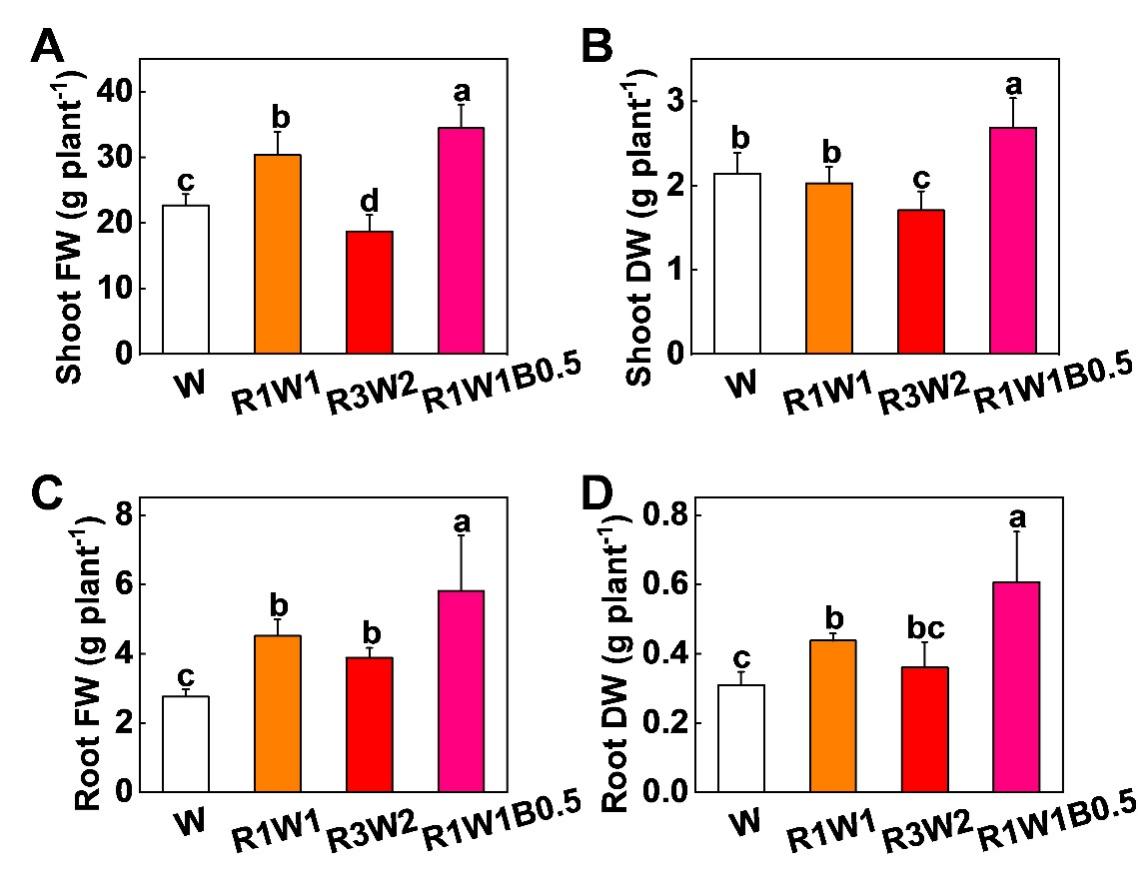


**Fig. S1.** **Various light environments influence biomass accumulation in tomato seedlings.** (A-D) The shoot fresh weight (A) and dry weight (B), and root fresh weight (C) and dry weight (D) in tomato plants grown under white (W), red-white (R1W1 and R3W2), and red-white-blue (R1W1B0.5) light conditions for 15 d. Values are the means of three biological replicates (±SD). Statistical significant is denoted by different letters.


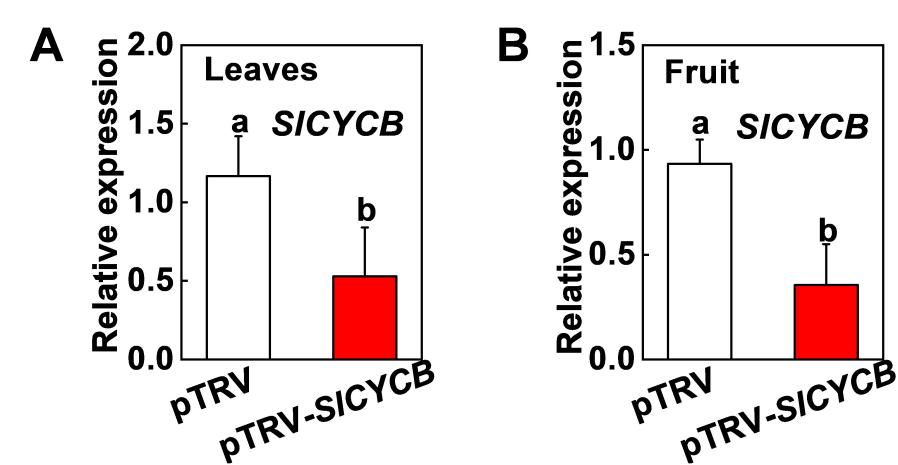


**Fig. S2.** **The relative expression of *SlCYCB* in the *SlCYCB-*silenced (pTRV-*SlCYCB*) and non-silenced (pTRV) tomato plants**. Samples are from the 4^th^ leaf (A) and tomato fruits at the mature green stage been infiltrated for 5 d later (B), respectively. Values are the means of three biological replicates (±SD). Statistical significant is denoted by different letters.


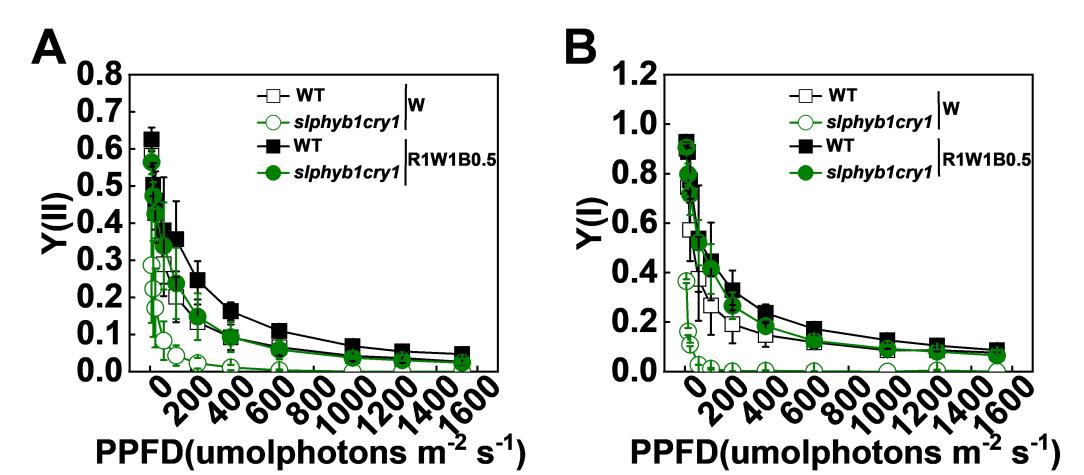


**Fig. S3. The effective quantum yield of PSII and PSI in *slphyb1cry1* mutant and wide-type (WT) plants.** (A-B) The effective quantum yield of PSII [Y(II); A] and PSI [Y(I); B] in *slphyb1cry1* mutant and wide-type (WT) plants cultured under white (W) and red-white-blue (R1W1B0.5) light conditions for 15 d. Values are the means of three biological replicates (±SD). Statistical significant is denoted by different letters.
