# Supplementary material for: Light quality regulates plant biomass and fruit quality through a photoreceptor-dependent HY5-LHC/CYCB module in tomato
Source: Hortic Res. 2023 Nov 16;10(12):uhad219. doi: 10.1093/hr/uhad219 (PMC10699845; doi:10.1093/hr/uhad219)
Supplement: Web_Material_uhad219 [file web_material_uhad219.zip › 2023.7.14.Supplemental Tables 1-3.docx]

Table S1 PCR primer sequences used for vector construction.

| **Vector** | **primer** | | |
| --- | --- | --- | --- |
| **SK-*SlHY5*** | | **Forward** | 5′-CGCggatccATGCAAGAGCAAGCGACGAG-3′ |
|  |  | **Reverse** | 5′-CCGgaattcCTTCCTCCCTTCCTGTGCAC-3′ |
| ***SlLHCAp:*LUC** | | **Forward** | 5′-GCCCCCCCTCGAggtcgacCGGGATTTCCTTTGGGCTACGTCAT-3′ |
|  |  | **Reverse** | 5′-CTCTAGAACTAGTggatccGCATCACCACTAGAAGTTCAAAAGA-3′ |
| ***SlLHCBp:*LUC** | | **Forward** | 5′-GCCCCCCCTCGAggtcgacCACGTGATATTATTTTTTTTACTTT-3′ |
|  |  | **Reverse** | 5′-CTCTAGAACTAGTggatccGACGTACAACTAAGCAGTTGCTGAT-3′ |
| ***SlCYCBp:*LUC** | | **Forward** | 5′-GCCCCCCCTCGAggtcgacCTTTGAGTTAATTGAAAAAAGTGAA-3′ |
|  |  | **Reverse** | 5′-CTCTAGAACTAGTggatccGTAGTCCACCACATCGACTAGTTTG-3′ |
| **pTRV2-*SlCYCB*** | | **Forward** | 5′-CTAGtctagaTGTGATGATGGTAAGAAGATAA-3′ |
|  |  | **Reverse** | 5′-CGCggatccATACGATAAAACAGGACGAC-3′ |

The restriction enzyme cutting sites were indicated in red.

Table S2 List of primer sequences used for qRT-PCR analysis.

| **Gene** | **Accession number** | **Forward primer (5’-3’)** | **Reverse primer (5’-3’)** |  |
| --- | --- | --- | --- | --- |
| ***SlHY5*** | Solyc08g061130 | GCAAGCGACGAGTTCTAT | ATCTCCGGCACTCTTCTG | |
| ***SlCYCB*** | Solyc06g074240 | GGGCTCAATTCGACGTGATC | CATGGAGAGTGGTGAAGGGT | |
| ***SlLHCB*** | Solyc08g067320 | GAATGGGAGACTTGCTATGTT | AACTGGATCAGCGATGTGGT | |
|  | Solyc09g014520 | CAACTCCGTTTCAGCCTTAC | CAACATAGCCCATCTTCCAT | |
|  | Solyc07g063600 | GCTCAGCCACAGTTGTTAGA | CAGGTCCATACCACAAATCA | |
|  | Solyc12g011450 | TGGGACACTGCTGGTTTATC | CACCGTCACTGAAGATTTGT | |
|  | Solyc07g047850 | TTTTCGGAGCAAACACCATC | GCACCAAGCATAGCCCAACG | |
|  | Solyc12g006140 | GGAGAAGATAGGCCAAAGTA | CAGTGTCCCATCCATAGTCA | |
| ***SlLHCA*** | Solyc12g009200 | AATCAGGGAGCTACCTACAA | CTTCTTTACCAGCATACAATCT | |
|  | Solyc05g056070 | GCTACTCTAAAGACCCTGCTAA | AAATCCCACAAATGCCAACA | |

**Table S3** Probes used in the EMSA**.**

| **Probes** | **Forward primer (5’-3’)** | **Reverse primer (5’-3’)** |
| --- | --- | --- |
| ***SlLHCA*** | CAAATTGGTGACAACGTACATTCTGGCA | TGCCAGAATGTACGTTGTCACCAATTTG |
| ***SlLHCA-m*** | CAAATTGGTGACAttttACATTCTGGCA | TGCCAGAATGTaaaaTGTCACCAATTTG |
| ***SlLHCB*** | CTGCTTAGTTGTACGTCTTGTAATCCTC | GAGGATTACAAGACGTACAACTAAGCAG |
| ***SlLHCB-m*** | CTGCTTAGTTGTttttCTTGTAATCCTC | GAGGATTACAAGaaaaACAACTAAGCAG |
| ***SlCYCB*** | ACGTGTTTATTTTTTACTTTAAGAACGTG | CACGTTCTTAAAGTAAAAAATAAACACGT |
| ***SlCYCB*-m** | ACGTGTTTATTTTTTACTTTAAGAttttG | CaaaaTCTTAAAGTAAAAAATAAACACGT |

The ACE-box and its mutant sites were indicated in red.
